# Shaping of Peripheral T Cell Responses by Lymphatic Endothelial Cells

**DOI:** 10.3389/fimmu.2016.00684

**Published:** 2017-01-12

**Authors:** Marion Humbert, Stéphanie Hugues, Juan Dubrot

**Affiliations:** ^1^Department of Pathology and Immunology, University of Geneva Medical School, Geneva, Switzerland

**Keywords:** lymphatic endothelial cells, peripheral tissue antigens, antigen presentation, immunomodulation, tolerance

## Abstract

Lymph node stromal cells (LNSCs) have newly been promoted to the rank of new modulators of T cell responses. The different non-hematopoietic cell subsets in lymph node (LN) were considered for years as a simple scaffold, forming routes and proper environment for antigen (Ag)-lymphocyte encountering. Deeper characterization of those cells has recently clearly shown their impact on both dendritic cell and T cell functions. In particular, lymphatic endothelial cells (LECs) control lymphocyte trafficking and homeostasis in LNs and limit adaptive immune responses. Therefore, the new role of LECs in shaping immune responses has drawn the attention of immunologists. Striking is the discovery that LECs, among other LNSCs, ectopically express a large range of peripheral tissue-restricted Ags (PTAs), and further present PTA-derived peptides through major histocompatibility class I molecules to induce self-reactive CD8^+^ T cell deletional tolerance. In addition, both steady-state and tumor-associated LECs were described to be capable of exogenous Ag cross-presentation. Whether LECs can similarly impact CD4^+^ T cell responses through major histocompatibility class II restricted Ag presentation is still a matter of debate. Here, we review and discuss our current knowledge on the contribution of Ag-presenting LECs as regulators of peripheral T cell responses in different immunological contexts, including autoimmunity and cancer.

## Introduction

The lymphatic system comprises a network of vessels together with lymphoid tissues all over the body that drain the extracellular compartment from most of the tissues. It transports lymph fluid, which is composed of immune cells and proteins drained from interstitial tissues, and helps to dispose of toxins and other unwanted components from the body. Lymphocytes follow the lymphatic system to migrate to infection sites, which supports and facilitates immune responses against potential harms. Frequently underestimated by scientists, the importance of lymphatics in controlling the immune system beyond the regulation of leukocyte trafficking has reached a new level with recent discoveries.

The initial observations of the lymphatic system date back to the Ancient Greece, referred to as “white blood.” However, it was in the seventeenth century that Asellius formally discovered the lymphatic vessels or, what he called, the “milky veins” from mesenteries in dogs (1). Several diseases have been described to result from failures in the lymphatic system, some of them having life-threatening consequences, such as lymphedema (2). Even more strikingly, the role of lymphatics in tumor spreading is known since the eighteenth century. Despite the ancient knowledge in the lymphatic system organization, our understanding in its multiple functions has rapidly evolved thanks to the unveiling of lymphatic endothelial cell (LEC) specific markers, such as the surface protein Lyve-1 or the transcription factor Prox-1, which are lacking in other endothelial cells. Several studies have subsequently demonstrated that LECs impact immune responses in many ways, including the modulation of immune cell migration and encounter, effector functions, and survival. In this review, we discuss our current understanding of the imunoregulatory properties of LECs. We specifically discuss the ability of LECs to directly impact T cell responses by presenting endogenous or exogenous antigens (Ags) to T cells in lymph nodes (LNs), and therefore to shape Ag-specific peripheral T cell responses in the context of autoimmunity and cancers.

## Origin and Types of Lymphatics

### LEC Development

Nowadays, it is well accepted and documented that, during embryogenesis, LECs differentiate from specialized angioblasts in the developing veins (3, 4). Nevertheless, this has been controversial for long until just few decades ago due to, in particular, the lack of knowledge on lymphatic-specific markers. Two different hypotheses raised in early twentieth century debated the possible origin of the lymphatic system. On one hand, studies on embryonic cats suggested that primary lymph sacs arised from mesenchymal progenitors (5). On the other hand, intravenous injection of ink in pig embryos revealed that lymph sacs developed from budding of embryonic veins (6, 7). The identification of the vascular endothelial growth factor receptor-3 (VEGFR-3) (8) reinforced the latter hypothesis of a common origin for both lymphatic and blood endothelial cells (BECs). In adulthood, VEGFR-3 expression is restricted to LECs (8, 9). However, it is also expressed by angioblasts and developing veins during embryonic development (8, 10, 11). Impaired development of both lymphatic and blood endothelium in VEGFR-3-deficient mice suggested a common progenitor for LECs and BECs (11). Further ratification of VEGFR-3 requirement for lymphatic development was provided by studies modulating the expression of its main ligand, the vascular endothelial growth factor C (VEGF-C). Overexpression of VEGF-C induced lymphatic sprouting and lymphangiogenesis (12–14).

The identification of the homeobox gene Prox-1 in 1993 led few years later to the final confirmation of the theory proposing the venous origin of lymphatics. Deletion of Prox-1 in mice results in the absence of early lymphatic endothelial differentiation and, as a consequence, Prox-1 knockout mice totally lack the lymphatic system (10, 15). Prox-1 expression in particular cells of the embryonic veins at E9.5 starts the lymphatic polarization and imprints the LEC signature (10, 15, 16). Transcriptome studies showed high proximity in LECs and BECs gene expression profiles. However, Prox-1 acts as the specific regulator of genes that are inversely regulated in a type-specific manner (17, 18). Indeed, potentially all venous endothelial cells may give rise to blood or lymphatic endothelium as demonstrated by Prox-1-induced reprograming when overexpressed in BECs (16). After development, functional Prox-1 is required to maintain the lymphatic phenotype (19). The molecular mechanisms of Prox-1-driven lymphatic differentiation have been reviewed recently (4). In addition, recent studies in zebrafish validated the molecular mechanisms governing lymphatic development, further demonstrating that the vast majority of cells contributing to LECs in thoracic ducts of zebrafish raised from primitive veins (3, 20). Later in development, however, the origin of organ-specific lymphatic vasculature might be slightly different. Using cell-fate mapping technologies, a recent publication suggested a combination of venous- and non-venous-derived LECs in the developing cardiac lymphatics (21). This spatiotemporal discrepancy may explain the difficulties experienced in obtaining a fully convincing explanation in the origin of LECs.

The specification of LECs during development entails structural and functional differences between blood and lymphatic systems. In sharp contrast to the circular and closed blood vasculature, lymphatic circulation appears as a linear- and blind-ended circuit. Capillaries of the lymphatic system drain interstitial fluids from peripheral organs and tissues thanks to the particular organization of LECs in the terminal lymphatics. The uptake of interstitial fluid, macromolecules, and cells is possible due to the highly permeable thin-walled capillary vessels composed of a single layer of LECs, which are not covered by pericytes or smooth muscle cells and have little or no basement membrane (22). Lymphatic capillaries exhibit discontinuous or “button-like” junctions where the interjunctional gaps act as sites of leukocyte entry into the vessels (23, 24). Terminal lymphatic capillaries are linked to the surrounding extracellular matrix by anchoring filaments that sense changes in interstitial pressures during inflammation. This results in vessel lumen and junction aperture, therefore facilitating the uptake of tissue-derived fluids. Deeper, lymphatics change from a drainage-prone phenotype to a collector vessel morphology specialized in lymph transport. Collecting lymphatics are surrounded by pericytes and smooth muscle cells and possess a basement membrane, displaying continuous “zipper-like” junctions. The presence of valves (22, 23) ensures the lymph circulation while preventing retrograde flow.

### Main LEC Types

Lymphatic vessels are present in almost all the vascularized organs, with the exception of the bone marrow. LEC immune modulatory properties represent a growing research area. LN LECs being the most characterized subset and representing the objective of this review is not discussed in this section.

However, lessons taken from studies performed during the last decade clearly establish different functions and possible roles for LECs from different anatomic locations. Deeper and careful future analyses will identify specific immunoregulatory features of distinct LEC populations.

For decades, lymphatic drainage was suggested to be involved in local immune responses (25). Dendritic cells (DCs) draw all the attention in initiating and eliciting tolerance or activation of the immune system. However, the role of lymph drainage in modulating adaptive immunity and tolerance remained largely unexplored. K14-VEGFR-3-Ig mice express soluble VEGFR-3-Ig *via* the keratin 14 promoter, resulting in a lack of lymphatic growth, which is restricted to the skin, and in a drop in fluid clearance (26). In these mice, local lymphatic drainage appeared to be critical for humoral immunity and acquired tolerance, while T cell responses remained delayed but mostly unaffected. There is no doubt that additional mechanisms and functions of dermal LECs will be discovered in the future.

LSECs could be seen as LEC counterparts in the liver. First described in 1970 (27), LSECs possess a high ability to filter fluids, solutes, and particles from hepatic circulation, occupy a large surface area exposed to blood that carries external food and commensal bacterial Ag, and are known to cross-present exogenous Ag to T cells (28).

A traditional dogma states the immune privilege and lack of lymphatic system in the central nervous system (CNS). This idea has persisted despite the notion of immune surveillance of T cells in the brain (29). A recent and elegant study identified for the first time the lymphatic vasculature in a specific area of the meninges lining the dural sinuses (30). The vessels express LEC-specific markers such as Lyve-1, Prox-1, or Podoplanin and drain the cerebrospinal fluid to deep cervical LNs. These findings provide new insights in the establishment and progression of some neurological diseases involving immune cell contribution, such as multiple sclerosis or Alzheimer’s. Moreover, CNS-resident stromal fibroblastic and endothelial cells were shown to guide antiviral CD8^+^ T cell responses in a model of virus-induced neuroinflammation (31). The production of CCR7 ligands CCL19 and CCL21 by CNS stromal cells was found critical for the induction of viral-specific T cell recruitment and the support of local T cell reactivation. Whether newly discovered CNS lymphatics (30) similarly contribute to neuroinflammatory immunopathologies remains to be determined.

Lymphatic development in the tumor microenvironment, known as tumor lymphangiogenesis, has been extensively studied. The participation of tumor lymphatics in the spread of the disease, or metastasis, has been studied for many years. In fact, most human melanomas and carcinomas metastasize through the lymphatic system (32). The presence of tumor-associated LECs correlates with bad clinical outcome in several types of cancer (33) and therapies aiming the blockade of tumor lymphangiogenesis are being considered for treatment of such malignancies (34). Growing evidence highlight the impact of tumor-associated LECs in dampening antitumor immunity. How interactions between lymphatics and T cells in the context of tumor development will further alter T cell responses is discussed below.

## Ag Presentation Independent Impact of LECs on Peripheral T Cell Responses

Hallmarks of T cell immunity include the generation of pathogen-specific effector responses to confer protection against a large range of invaders, without causing unwanted self-tissue damage. Naïve T cells constantly scan for their cognate Ag. However, given the extremely low frequency of T cells being specific for a particular peptide–major histocompatibility (MHC) complex (35, 36), this challenging task is strictly located into highly organized secondary lymphoid organs (SLOs), such as LNs, Peyer’s patches (PPs), and the spleen. These SLOs contain both tissue-derived and blood-borne Ags, therefore facilitating naïve T cell-Ag encounter, and subsequent T cell activation and differentiation into T cell effectors. This part summarizes the different pathways by which LECs will impact T cell outcome inside and after exiting LNs.

### Ag Delivery to LNs

As described before, LNs are connected to lymphatics, which drain peripheral tissue-derived fluids. By connecting tissues to draining LNs, LECs facilitate the passive entry of tissue-derived Ags that can thereby be captured, processed, and presented by resident DCs to T cells entering LNs through high endothelial venules (37, 38). Soluble Ags are immediately sampled by LN DCs, whereas particles carrying Ags, such as exosomes, apoptotic bodies or microvesicles, which have not been captured by subcapsular sinus macrophages, flow to LN medullary sinuses where they can be sampled by DCs (39). LECs also support the active migration of tissue-resident DCs into LNs. DC migration from tissues to draining LNs *via* lymphatic vessels is an important way to present Ags and activate naïve T cells. DCs enter afferent lymphatics through preformed portals (40), independent of integrin-mediated adhesion (41). However, LECs upregulate adhesion molecules upon inflammation, further favoring DC access to lymphatic vessels (42). In addition, expression of CLEC2 (a C-type lectin receptor) by DCs promotes their migration to LNs *via* lymphatics through interaction with its ligand gp38 (Podoplanin), which is expressed by both LECs and fibroblastic reticular cells (FRCs) (43).

### Modulation of DC Functions

Tissue-resident DCs having acquired peripheral Ags subsequently migrate through afferent lymphatics into LNs in a CCR7-dependent manner. However, the lymphatic system does not only support DC migration from tissues to LNs. Indeed, close interactions between migrating DCs and LECs induce phenotypic and functional changes in DCs. First, contacts between TNF-α-stimulated LECs and DCs lead to decreased expression of costimulatory molecules by DCs *in vitro*, thus impairing DC ability to induce T cell proliferation (44). LEC-mediated regulation of DC functions is dependent on interactions between CD11b (Mac-1) on DCs and ICAM-1 on LECs (44). Interestingly, LECs are able to inhibit the function of LPS-activated DCs, suggesting once again a regulatory role for LECs in the resolution phase of inflammation. A recent report demonstrated that LECs function as reservoirs of peripheral tissue-restricted Ags (PTAs), which are subsequently acquired and presented by DCs to induce T cell anergy, therefore contributing to peripheral CD4^+^ T cell tolerance (45).

### T Cell Homeostasis

While T cell migration inside LNs is mainly driven by CCL19 and CCL21 produced by FRCs (46), naive and memory T lymphocyte maintenance in SLOs is highly dependent on IL-7. Together with FRCs (47), LECs represent an important source of IL-7 *in vivo*, regulating lymphocyte homeostasis and access to SLOs. IL-7-GFP knock-in mice exhibit moderate GFP expression in LN-FRCs, whereas high levels were detected in both LN LECs and tissue LECs (48, 49). Similarly, LECs were shown to be the major source of IL-7 in both human and murine LNs (50). Furthermore, LECs not only produce IL-7 but also express the IL-7 receptor chains IL-7Rα and CD132, suggesting a possible role for IL-7 as an autocrine mediator of lymphatic drainage. IL-7-stimulated LECs induced lymphangiogenesis in the cornea of mice *in vitro*, whereas in IL-7Rα^−/−^ mice, lymphatic drainage was compromised (51). In addition, IL-7 upregulation by both FRCs and LECs is essential for LN reconstruction and remodeling following viral infection or avascular transplantation (50). This suggests that IL-7 production in LN after resolution of an infection could be involved in memory T cell homeostasis. Accordingly, IL-7 promotes the development, the proliferation, and the survival of memory CD8^+^ T cells (52, 53).

### T Cell Egress from LNs

T cell egress from LNs is dependent on their expression of the sphingosine-1-phosphate (S1P) receptor (S1PR1). Using mice lacking S1P selectively in LECs while maintaining normal blood S1P, Cyster and collaborators have shown that LECs are an *in vivo* source of S1P in LNs, allowing T cell egress from LNs and PPs (54). S1PR1 expression is downregulated by blood circulating lymphocytes, and upregulated in LNs. Interactions between S1P-producing LECs and S1P1R-expressing T cells promote LN egress by overcoming retention signals mediated by CCR7 (55, 56). Although steady-state LECs express low levels of S1P, its production is upregulated in medullary sinus LECs upon PAMP/DAMP-mediated inflammation, suggesting that high S1P-expressing LECs can promote effector T cell egress from LNs in pathogenic situations. In contrast, in non-infectious sterile inflammatory contexts, low S1P-producing LECs would rather dampen T cell effector functions by favoring T cell retention in LNs.

### T Cell Migration in Tumor-Associated Lymphatics

Increasing evidence suggest that tumor-associated lymphatics not only simply function as tumor cell transporters but also play additional important roles impacting tumor development. Accordingly, not only metastatic but also primary tumor progression can be affected by modulating tumor-associated lymphatic expansion. In the context of solid tumors, lymph flow from tumors is elevated, driving intense interstitial flow in the tumor stroma and increasing lymphatic drainage from the tumor to the draining LN (57). Combined with a suppressive cytokine environment, it is therefore possible that increased tumor Ags drainage could promote tumor-specific T cell dysfunction, including anergy and apoptosis. In addition, the lymph supports cells migrating from tissues, in particular CCR7^+^ DCs, a phenomenon shown to be critical for initiating antitumor immune responses (58).

Tumor infiltration by T cells is one of the key steps in antitumor immunity. While cytotoxic T lymphocyte infiltration correlates with good prognosis, accumulation of T regulatory cells (Treg) or naïve T cells is detrimental for the clinical outcome (59, 60). Likewise, expression of CCL21 in the tumor promotes immune escape and tumor progression (61), which may be explained, at least in part, by the enhancement of naïve T cell recruitment. Although T cell receptor (TCR)-transgenic tumor-infiltrating naïve T cells may be activated *in situ* (62), it is unlikely, given the immunosuppressive tumor-related environment, that this will lead to fully competent effector T cell differentiation. In this regard, it is still to be demonstrated whether CCL21-producing LECs contribute to this effect. How LECs contribute to the overall tolerogenic properties of the tumor microenvironment is still an open question.

We have demonstrated that the lymphangiogenic growth factor VEGF-C produced in the tumor promoted immunological tolerance in murine melanoma (63). VEGF-C protected tumors against preexisting antitumor immunity and promoted local deletion of tumor-specific CD8^+^ T cells (63, 64). Our findings introduce a new role for lymphatics in promoting tumor development and suggest that lymphatic endothelium in the local microenvironment may be a novel target for immunomodulation. Supporting those hypotheses there is a recent publication demonstrating that following exposure to tumor-derived factors, FRCs of the tumor-draining LNs adapt on multiple levels to exhibit features associated with immunosuppression, such as decreased production of IL-7 and CCL19/21 (65). Whether a similar profound reprograming occurs to LECs in tumor-draining LNs remains to be determined.

## Ag Presentation-Dependent Impact of LECs on Peripheral T Cell Responses

In addition to their ability to modulate T cell responses by impacting immune cell migration, interactions, and homeostasis, LECs can also function as Ag-presenting cells through several mechanisms and directly influence peripheral T cell outcome.

### Presentation of Endogenously Expressed PTAs to T Cells by LECs

**Figure 1:**
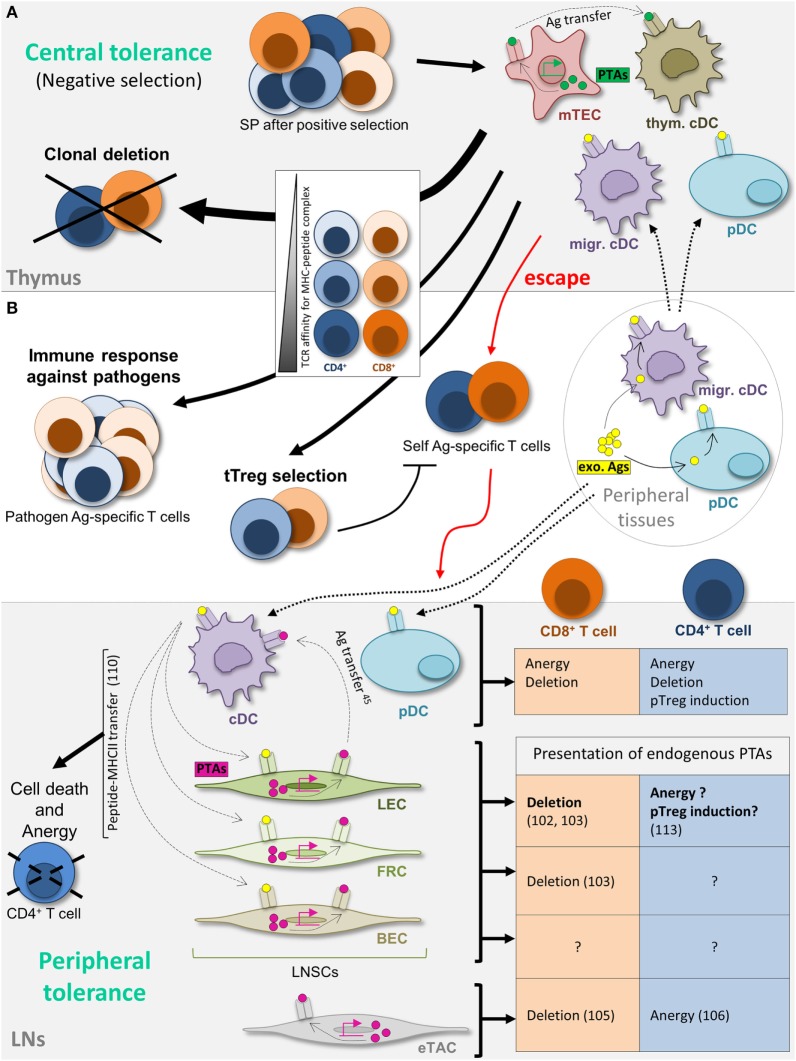
**Maintenance of T cell tolerance**. **(A)** Schematic view of thymic central tolerance, reviewed in Ref. (67). After positive selection (not depicted), simple positive (SP) thymocytes undergo a process of negative selection. Thymus-resident conventional dendritic cells (cDCs) and peripheral tissue-restricted antigens (Ags) (PTA) (green)-expressing medullary thymic epithelial cells, as well as peripheral plasmacytoid DCs (pDCs) and cDCs, that have acquired Ag (yellow) in the periphery and migrate to the thymus, present self-peptide major histocompatibility complex (MHC) complexes to SP thymocytes. Thymocytes expressing a T cell receptor (TCR) with high affinity for self (dark colors) are clonally deleted. SP expressing a TCR with intermediate affinity differentiate into thymus-derived T regulatory cell (tTreg) (medium colors). Low-affinity TCR-expressing SP (light colors) exit from the thymus and enter the periphery, however comprising some self-reactive T cells (dark colors) that escaped central tolerance. **(B)** Peripheral T cell tolerance in the lymph nodes (LNs). References related to lymph node stromal cell contributions are indicated (numbers). Self-Ag-specific T cell tolerance is further maintained in the periphery in LNs. cDCs and pDCs acquire Ag from peripheral tissues (yellow) and migrate to LNs to present Ag to autoreactive T cells. cDCs also acquire Ag expressed by lymphatic endothelial cells (LECs). LECs, fibroblastic reticular cells, and blood endothelial cells present endogenously expressed PTAs (pink), as well as peptide–MHC-II complexes acquired from cDCs, therefore contributing to peripheral T cell tolerance *via* distinct mechanisms. Extrathymic autoimmune regulator (Aire)-expressing cells (eTACs) present endogenously expressed PTAs. The outcome of Ag presentation by each cell subtype is depicted in the figure. Cell migration and Ag transfer are represented by dotted and dashed arrows, respectively. exo Ags, exogenous antigens; migr. cDC, migratory cDC; pTreg, peripherally induced Treg; thym. cDC, thymus-resident cDC.

In order to prevent autoimmunity, thymocytes go through a process of negative selection, part of the so-called central tolerance, allowing the deletion of autoreactive T cell clones before they exit from the thymus to enter into the periphery [reviewed in Ref. (66, 67)]. In the thymus, medullary thymic epithelial cells (mTECs) promiscuously express PTAs, Ag that are normally expressed in the periphery (68, 69). The expression of a vast majority of PTAs in mTECs is regulated by transcription factors (70), including the autoimmune regulator (Aire), mutations in Aire leading to severe autoimmune disorders (71, 72). PTAs can be either directly presented by mTECs to the thymocytes, acquired from mTECs by thymus-resident DCs or acquired in tissues by migrating DCs or plasmacytoid DCs (pDCs), and cross-presented to the thymocytes (73–76) (Figure 1A). Thymocytes expressing a TCR with a too high affinity for self-Ag/MHC complexes undergo clonal deletion (73–75). A fraction of the CD4^+^ thymocytes having a TCR with a high affinity differentiates into thymus-derived Tregs (tTregs), previously called natural Tregs (nTregs), and expresses the transcription factor Foxp3 (77). A population of CD8^+^ Foxp3^+^ tTregs has also been described (78–81). However, some autoreactive—non-Treg—T cells do escape thymic central tolerance mechanisms and reach the periphery (82, 83), as a result from either an absence of specific self-Ag presentation in the thymus, or a lack of deletion due to a TCR exhibiting an affinity for self-Ag/MHC complexes below the negative selection threshold (84) (Figure 1A).

Therefore, additional mechanisms, called peripheral tolerance mechanisms, have evolved to maintain T cell tolerance apart from the thymus [reviewed in Ref. (66, 85)]. Cross-tolerance induction by peripheral DCs has been extensively studied and reviewed over the past two decades (86); immature DCs acquire Ag through the phagocytosis of apoptotic cells in peripheral tissues to present them to T cells in SLOs (87–89). In the absence of costimulatory signals, Ag presentation leads to CD4^+^ and CD8^+^ T cell clonal deletion (physical elimination) or anergy (functional inactivation) and/or to the induction of peripherally induced Tregs (pTregs), previously called induced Tregs (iTregs) in the presence of anti-inflammatory factors (77, 90–92). Both resident and migratory DCs, including pDCs, contribute to this process in the LNs (93–96) (Figure 1B). Nevertheless, emerging evidence demonstrates that peripheral tolerance does not exclusively rely on DCs. Lymph node stromal cells (LNSCs), and in particular LECs, also play an important role in the maintenance of peripheral tolerance (Figure 1B).

### PTA-specific Expression by LECs

The discovery of the ectopic PTA expression by mTECs in the thymus was the first example that cells of non-hematopoietic origin present endogenously expressed self-Ag to T cells (68, 69). Using GFAP-HA or iFABP-tOVA transgenic mouse models, in which hemagglutinin (HA) or a truncated form of ovalbumin (tOVA) are expressed as self-Ag in enteric glial cells (EGCs) or mature intestinal epithelial cells (IECs), respectively, it was shown few years ago that the EGC-associated HA or IEC-associated tOVA proteins were unexpectedly expressed not only by EGCs or IECs but also by CD45-negative stromal cells, in all LNs and not exclusively in mesenteric LNs. Those LNSCs were able to process endogenously expressed self-proteins into antigenic peptides to directly present these Ag to CD8^+^ T cells in SLOs, making them functionally similar to mTECs in the thymus (97–100). Moreover, it was shown in non-transgenic mouse models that LNSCs naturally express PTAs and directly present them to CD8^+^ T cells. Among other examples, LNSCs ectopically express tyrosinase (tyr), while its expression is normally confined to melanocytes (101). It was later shown that LECs are the only cells ectopically expressing this Ag in the LN (102, 103). Indeed, using CD31 and gp38 (Podoplanin) as markers to distinguish the LNSC subtypes, it was observed that each subtype expresses a distinct set of PTAs, with some PTAs exclusively expressed in one specific LNSC subset and some others redundantly expressed (102, 103) (Figure 1B). This suggests a non-redundant role for the different LNSC subtypes in the tolerization of various self-specific T cells. In addition, the expression of PTAs by LECs is subanatomically compartmentalized, with a high expression of PTAs observed only in LN medullary sinus LECs (104).

In mTECs, the expression of most, but not all, PTAs is regulated by Aire (70, 71). In the LN, a rare bone marrow-derived population was described to express Aire and was called extrathymic Aire-expressing cells (eTACs). Consequently, eTACs express various PTAs in an Aire-dependent manner, and present them through major histocompatibility complex class I (MHC-I) and MHC-II molecules to induce CD8^+^ T cell deletion (105), and CD4^+^ T cell anergy (106), respectively (Figure 1B). On the contrary, PTAs expressed by non-hematopoietic LNSCs, including LECs, are not dependent on Aire (103). The regulation of the expression of the pancreatic self-Ag Ppy by LECs in pancreatic LNs depends on the transcriptional regulator Deaf1, which, together with Aire, belongs to the SAND gene family (107, 108). Interestingly, variant isoforms of Deaf1 in mice and human display an impaired Ppy expression, and were linked to autoimmune type I diabetes (107). The fact that LNSCs do not express Aire may explain the low overlapping PTA expression in mTECs and LNSCs (109), therefore suggesting a complementary contribution of mTECs and LNSCs in T cell tolerance induction and maintenance. Future investigations will identify other transcription factors, selectively or commonly expressed by LNSC subsets, which promote different PTA expression.

### PTA Presentation by LECs to T Cells

LNSCs not only endogenously express PTAs but also the direct presentation of PTA-derived peptides in the context of MHC-I molecules to CD8^+^ T cells leads to their clonal deletion and subsequent tolerance induction (97, 98, 101) (Figure 1B). In the GFAP-HA or iFABP-tOVA models mentioned above, the lack of presentation of HA or tOVA by enteric stromal cells to HA- or tOVA-specific CD8^+^ T cells was associated with enteric autoimmunity. Among other LNSC subsets, LECs are involved in this CD8^+^ T cell deletional tolerance and are necessary and sufficient for the induction of peripheral tolerance to some self-Ag, like Tyr, an autoantigen associated with autoimmune vitiligo (102, 103, 107). These studies show a crucial role for LECs in the maintenance of peripheral tolerance.

Nevertheless, the ability of LNSCs, and in particular LECs, to directly present endogenously expressed PTAs in the context of MHC-II molecules to CD4^+^ T cells is still a matter of debate, as well as the subsequent impact on CD4^+^ T cell outcome. We have previously shown that the endogenous expression of MHC-II molecules is regulated in LECs, BECs, and FRCs by the promoter IV (pIV) of the master regulator CIITA (110). One study has however demonstrated that the adoptive transfer of HA-specific TCR transgenic CD4^+^ T cells (6.5) in GFAP-HA transgenic mice, in which HA is expressed as an autoantigen by EGCs, did not dampen lethal enteric autoimmunity (98). However, as mentioned by the authors, the absence of direct presentation of HA peptide by LNSCs to HA-specific CD4^+^ T cells in their model does not rule out a possible upregulation of MHC-II molecules in LNSCs and a direct presentation under pro-inflammatory conditions (98). Indeed, several studies that will be discussed later in this review have suggested that LNSCs, among which LECs, upregulate MHC-II molecules at their surface upon inflammation (110, 111).

For their part, Engelhard and colleagues claim that LECs are unable to present endogenously expressed PTAs (β-galactosidase, membrane-bound HA or I-Eα in their models) to CD4^+^ T cells, not related to Ag localization but due to a lack of H2-M expression in LECs, which would prevent the loading of peptides onto MHC-II molecules (45). However, this study was carried out in the steady state, whereas LECs, BECs, and FRCs, that express IFN-γ inducible-CIITA pIV, might require IFN-γ to upregulate H-2M molecules, as they do for MHC-II expression, these two genes being co-regulated by CIITA (112). Moreover, Mebius and colleagues observed the presence of mRNA transcripts for H2-M in LECs, among other MHC-II-related molecules (113).

Mebius and colleagues identified that in transgenic mice expressing OVA under the control of the keratin 14 promoter (K14mOVA mice), OVA was unexpectedly expressed in LECs. In addition, OVA^+^ LEC were able to present OVA peptides through MHC-II to OTII cells *in vitro*, leading to an increased Foxp3^+^ OT-II cells Treg homeostasis (113). Using LN transplantation experiments, the authors further suggested that the presentation of endogenously expressed self-Ag by LNSCs, and especially by LECs, contribute *in vivo* to the maintenance of Foxp3^+^ CD4^+^ Tregs in the periphery (Figure 1B) (113). Finally, lentiviral vectors allowing the selective transduction of MHC-II^+^ non-hematopoietic cells with MHC-II- and MHC-I-restricted HY male-derived epitopes induced T cell hyporesponsiveness/anergy of HY-specific CD4^+^ and CD8^+^ T cells in female mice (114). Moreover, in Marilyn TCR transgenic mice expressing HY-specific CD4^+^ T cells, increased conversion of effector CD4^+^ T cells into CD25^+^ Foxp3^+^ pTregs was observed (114). Whether these effects were due to a direct Ag presentation of endogenously expressed HY to CD4^+^ T cells by gp38^+^ stromal cells, i.e., LECs and FRCs in the LN, remains to be determined. Indeed, as stated by the authors, they cannot rule out that other, non-DC, hematopoietic cell types could contribute to the presentation of HY Ags, due to undesired transduction and subsequent direct Ag presentation and/or Ag transfer to stromal cells (110, 114). Despite a lack of demonstration of direct Ag presentation by gp38^+^ stromal cells and the lack of distinction between the contribution of the different stromal cell subtypes in this model, these data are in accordance with the results of Baptista et al., as mentioned above (113).

### Molecular Pathways Involved in LEC-Mediated Peripheral T Cell Tolerance

The molecular pathways involved in the clonal deletion of CD8^+^ T cells by LNSCs, and in particular by LECs, are not fully elucidated. Using the iFABP-tOVA transgenic mouse model described above, in which tOVA is expressed as a self-Ag in the intestinal epithelium, it was shown that the induction of CD8^+^ T cell tolerance requires PD-1:PD-L1 interaction, as the disruption of this pathway leads to severe intestinal enteric autoimmune disorder (115). More specifically, in a model of adoptive transfer of Tyr-specific TCR transgenic CD8^+^ T cells (FH T cells) into Tyr-expressing bone marrow chimeric mice, in which either radiosensitive hematopoietic or radioresistant non-hematopoietic cells lacked PD-L1 expression, FH T cells were deleted only when PD-L1 was expressed by the non-hematopoietic LN compartment (116). Moreover, among the LNSC subsets, LECs were the ones expressing the highest level of PD-L1, with medullary sinuses LECs being the highest expressers. In addition, LECs do not express costimulatory molecules at their surface. The administration of agonistic anti-4-1BB antibodies prevented the deletion of FH CD8^+^ T cells. The lack of costimulation through 4-1BB by LECs would lead to PD-1 upregulation by FH T cells, as Tyr presentation by LECs led to a higher expression of PD-1 by FH T cells, an effect that was suppressed upon agonistic anti-4-1BB antibody administration. This would, in turn, prevent CD25 upregulation, which is necessary for CD8^+^ T cells survival. Indeed, CD25 expression on FH T cells was upregulated only in the presence of agonistic anti-4-1BB or blocking anti-PD-L1 antibodies after Tyr presentation by LECs (116). Hence, in this model, LECs are responsible for the presentation of the endogenously expressed Tyr, which, together with a combination of a lack of costimulation and a provision of co-inhibitory signal, leads to Tyr-specific CD8^+^ T cell deletion (116). The high expression of PD-L1 in LECs is likely regulated by lymphotoxin β receptor (Ltβr), as the treatment of mice with anti-Ltβr antibodies led to decreased PD-L1 expression in LECs (104). Using μMT^−/−^, CD3ε^−/−^, and Rag1^−/−^ mice, it was further shown that B cells are required for the expression of the adhesion molecule MadCAM-1 at the surface of LECs in the medulla, itself necessary for the expression of PD-L1. On the contrary, T cells seemed to suppress PD-L1 expression in LECs through mechanisms that have not been deciphered yet (104). Finally, it was suggested that the expression of MHC-II on LECs would be involved in the induction of CD8^+^ T cells tolerance to endogenously expressed self-Ag in LECs by engaging the inhibitory molecule LAG-3. Indeed, after adoptive transfer of β-gal-specific TCR transgenic CD8^+^ T cells (Bg1 cells) into Prox-1xβgal mice, in which β-gal is selectively expressed by LECs, the proliferation of Bg1 cells was increased following administration of blocking anti-LAG-3 antibodies, which was acting in synergy with anti-PD-L1 blocking antibodies (45).

We previously showed that high PD-L1 expression by LECs correlate with their unique ability, compared to other LNSC subsets, to induce CD4^+^ T cell apoptosis after presentation of DC-acquired peptide–MHC-II complexes (110). Although the molecular mechanisms accounting for the induction of tolerance to MHC-II-restricted self-Ag endogenously expressed and directly presented by LECs to CD4^+^ T cells have not been elucidated so far, they are thus likely to involve PD-L1 expression by LECs, as in the case of CD8^+^ T cells.

### Ag Acquisition and Presentation by LECs to T Cells

**Figure 2:**
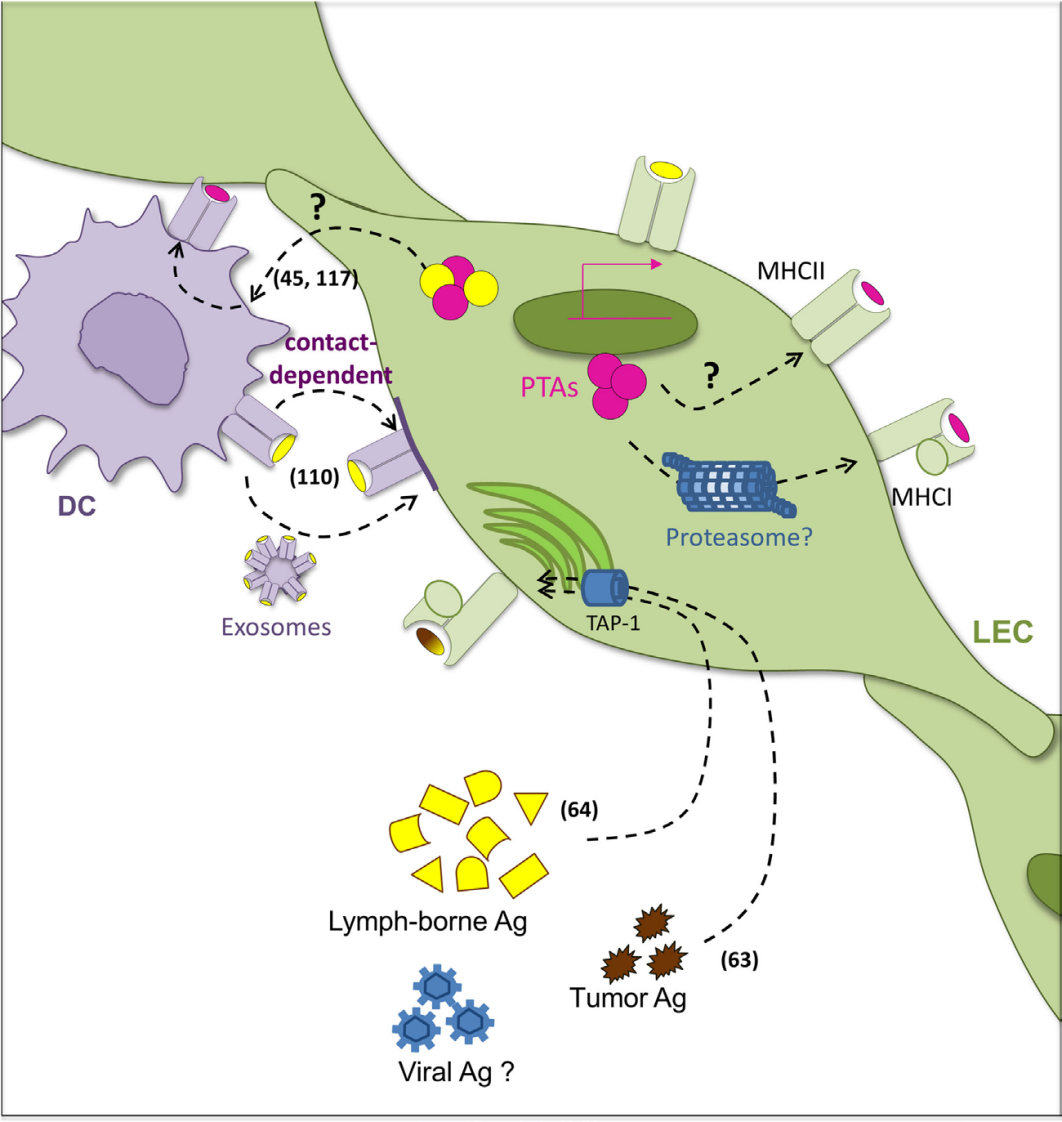
**Pathways of Ag acquisition and presentation by LECs**. Several pathways of antigen (Ag) acquisition and loading coexist in lymphatic endothelial cells (LECs). Interactions with dendritic cells (DCs) underlie complex mechanisms of Ag transfer in both directions. On one hand, LECs act as Ag reservoirs for DCs which can uptake LEC-derived Ag. The mechanisms accounting for this phenomenon remain however unclear. On the other hand, LECs acquire peptide–MHC-II complexes from DCs in a cell–cell contact dependent manner (DC-derived Ag is depicted in yellow). DC-derived exosomes might also be implicated. Peripheral tissue-restricted Ags (PTA) (in pink) expressed by LEC can be loaded into MHC-I molecules. Intracellular pathways of degradation of such PTAs have however been not investigated. Moreover, whether PTA can be incorporated in MHC-II compartments is still a matter of debate. Alternatively, LECs possess the ability to uptake exogenous lymph-borne and tumor-derived Ag that can be incorporated in MHC-I pathway in a TAP-1-dependent manner. Related references are indicated in numbers.

The lymphatic system, by controlling Ag availability, constitutes one of the first checkpoints for immune responses (100). It is not surprising then that LECs, which have early access to any given Ag, display different mechanisms for Ag uptake and processing (Figure 2). Indeed, recent work revealed that Ag trafficking can be observed at more levels than the classical concept of LECs as lymph carriers. Complex interactions between LECs and DCs (45, 110, 117) depict an exciting picture of Ag bidirectional exchange that ultimately may serve to modulate the overall magnitude of the immune response (Figure 2).

### Uptake of Exogenous Ag

It has been extensively demonstrated in several mouse and human models that LECs exhibit an active endocytotic capacity (38, 118). They are able to uptake exogenous molecules and, depending on their location, process Ag for cross-presentation and cross-priming of Ag-specific CD8^+^ T cells (63, 64) (Figure 2). Interestingly, Ag-loaded primary LN LECs were shown to be capable of cross-priming Ag-specific CD8^+^ T cells in a TAP1-dependent manner (64). As described above for endogenous PTA presentation, Ag-loaded LECs induced T cell apoptosis, the lack of expression of costimulatory molecules being the most extended explanation. LECs neither express nor upregulate the costimulatory molecules CD40, CD80, and CD86 following TLR engagement or in presence of IFN-γ or TNF-α (110, 116). While LECs upregulate the immunostimulatory molecules HVEM, CD48, and MHC-II under such conditions (116), they also upregulate PD-L1 (102, 110, 119). Pointing at the same direction, Ag cross-presentation by LSECs induces tolerized CD8^+^ T cells in the liver. In this context, PD-L1 expression was also relevant for such outcome (120). Interestingly, in the absence of inflammation, surviving LSEC-educated T cells had an Ag-experienced central memory-like phenotype in SLOs (121). Furthermore, LSEC-primed memory T cells could be reactivated *in vitro* and *in vivo* in an Ag-specific manner, and they could contribute to a viral challenge (121).

The direct contribution of Ag presentation by LECs to CD4^+^ viral immunity is still a matter of debate. As mentioned above, LECs serve as Ag reservoir during viral infections (117) (Figure 2). Nonetheless, genetic ablation of MHC-II in radioresistant stromal cells in LNs resulted in longer maintenance of Ag-specific CD4^+^ T cells (122). Specific impact of LN LECs and mechanisms accounting for such effects should be yet clarified.

### Cellular Ag Transfer

The hallmark of professional APCs is the constitutive cell surface presence of MHC-II and their ability for Ag processing and presentation (123). Constitutive MHC-II expression is restricted to a small number of cells of the immune system. Nonetheless, there are many different cell types from both hematopoietic and non-hematopoietic origins that can indeed express MHC-II and interact with CD4^+^ T cells in the periphery (100, 124, 125).

As mentioned above, LECs constitute such non-professional APC cell types that express MHC-II in an IFN-γ-dependent manner. Indeed, MHC-II expression in LN LECs has been reported at both transcriptional and protein expression levels (102, 110, 111). By using transgenic mouse models lacking the different CIITA promoters, we have previously demonstrated that steady-state levels of MHC-II molecules on the surface of LECs and other stromal subsets in LNs reflect a combination of IFN-γ-inducible basal activity and acquired peptide:MHC-II complexes from DCs (110). The acquired MHC-II molecules were loaded with DC-derived Ags, licensing LECs to induce anergy and increased cell death Ag-specific CD4^+^ T cells (Figures 1B and 2). Lack of measurable productive T cell responses has been one of the major difficulties preventing the clarification of the impact of Ag presentation by LECs on CD4^+^ T cell outcome. As for CD8^+^ T cell responses, the absence of costimulatory signals, such as CD80 or CD86 and the constitutive expression of PD-L1 by LECs, preclude the possibility of functional effector CD4^+^ T cell priming. In this regard, it has been shown that human LN-derived LECs fail to induce allogeneic CD4^+^ T cell proliferation even after IFN-γ stimulation (119). In these particular *in vitro* settings, LECs were unable to induce proliferation of either naïve or memory CD4^+^ T cells.

Membrane exchange between cells is not uncommon in immunology (126). Peptide:MHC-I and MHC-II complexes have been shown to be transferred between DC and tumor cells (127) or infected cells (128), as well as between DCs (129). Ag transfer can occur as peptide exchange on cell surfaces. Peptide epitopes can bind directly on cell surface or early endosomal MHC molecules (130), where both MHC-I and MHC-II are receptive for lymph-borne peptide binding. This might be particularly relevant in the context of self-tolerance, since recent analyses showed that the human lymph peptidome contains predominantly self-peptides, including products derived from extracellular processing of proteins (131). Exosomes were also implicated in the transfer of peptide:MHC-II complexes from DCs to LNSCs (110), and they cannot be excluded to contribute to alternative Ag trafficking (Figure 2).

Antigen transfer between LECs and DCs is, however, not restricted to one direction. Indeed, the transfer of PTAs specifically expressed in LECs to hematopoietic cells has been described (45) (Figure 2). Neither membrane-bound nor cytoplasmic PTAs were directly presented by LECs to prime Ag-specific CD4^+^ T cell responses. As mentioned above, this was attributed to the lower expression of H2-M in LECs compared to professional APCs, which is required for peptide binding into the MHC-II groove. Instead, peptides derived from PTAs expressed by LECs were found to be loaded onto MHC-II in DCs (45). While the exchange mechanism is still open to examination, it was reported not to be dependent on recognition of apoptotic cells or DC phagocytosis. These complementary bidirectional observations highlight the close relationship and communication between professional APCs and LECs to enable MHC-II presentation.

## Concluding Remarks

Increasing evidence suggest that lymphatics are much more than simple pipes that drain tissue-derived fluids containing proteins, particles, and cells. Through the expression of different surface molecules and the production of soluble factors, LECs indeed modulate immune responses in many ways, including the active regulation of cellular migration, interactions, and functions. Recent studies have highlighted a possible role for LECs as direct instructors of T cell immunity. Indeed, the discovery that LNSCs, including LECs, ectopically express tissue-derived Ags, a feature thought to be restricted to mTECs and thymic central T cell tolerance, has pushed forward LECs to potentially function as Ag-presenting cells. Accordingly, the selective expression of model Ags in LECs leads to an Ag-specific recognition by T cells, which, after an early step of activation and proliferation, are either inactivated or deleted. Therefore, the presentation of endogenously expressed Ags by LECs seems to contribute to peripheral T cell tolerance. Studies have also suggested that LECs acquire exogenous Ags by distinct pathways, including direct uptake, or cell-membrane transfer, and present them to induce T cell dysfunction. The molecular mechanisms contributing to LEC ability to inactivate T cells are still not fully elucidated. However, a consensus candidate, PD-L1, the ligand for program-cell death 1 receptor expressed by T cells, emerged from several recent studies to be highly expressed by LECs, and important to mediate T cell tolerance. Although pioneering studies suggest that Ag-presenting LNSCs are sufficient to maintain peripheral T cell tolerance, the specific contribution of LECs remains to be addressed. Likewise, substantial differences among LECs from distinct anatomical locations entail different functions. Specific roles of local LECs should be carefully dissected in order to fully understand how they differentially impact T cell responses. In addition, most studies so far have been performed in steady state, and the contribution of Ag presentation by LECs under different pathological conditions in shaping of peripheral T cell responses remains to be determined. In addition, future studies will assess how current therapies for cancer or autoimmune diseases aiming at modulating immune cell functions, specifically alter the ability of LECs to impact T cell responses.

## Author Contributions

SH, JD, and MH have developed the concept, wrote the manuscript, prepared the figures, and critically read, revised, and approved the manuscript.

## Conflict of Interest Statement

The authors declare that the research was conducted in the absence of any commercial or financial relationships that could be construed as a potential conflict of interest.
